# Gangrenous Digital Ischemia Unmasking HIV Infection in Untreated Systemic Sclerosis

**DOI:** 10.7759/cureus.98182

**Published:** 2025-11-30

**Authors:** Parto Siavosh, Jaafar Al-Sadiq Jaf, Alireza Hejrati, Parnasadat Hosseini Migoni, Ali Shbeeb

**Affiliations:** 1 Hematology and Medical Oncology, Rasoul Akram Hospital, Tehran, IRN; 2 Stroke, Stepping Hill Hospital, Stockport, GBR; 3 Department of Internal Medicine, School of Medicine, Hazrat-e Rasool General Hospital, Iran University of Medical Sciences, Tehran, IRN; 4 Department of Orthopedic Surgery, Joint Reconstruction Research Center, Imam Khomeini Hospital Complex, Tehran University of Medical Sciences, Tehran, IRN

**Keywords:** digital gangrene, hiv, ischemia, scleroderma, systemic sclerosis, vasculopathy

## Abstract

We report the case of a 55-year-old woman with untreated systemic sclerosis who presented with severe digital ischemia and dry gangrene requiring toe amputation. Further investigation revealed generalised lymphadenopathy and an unexpected diagnosis of HIV infection. Lymph node biopsy revealed reactive inflammatory changes associated with systemic sclerosis.

This case highlights the importance of evaluating coexisting infections in autoimmune diseases and the potential for severe vascular complications when scleroderma remains untreated, particularly in immunocompromised patients.

## Introduction

Systemic sclerosis (SSc) is a chronic autoimmune connective tissue disease characterised by immune dysregulation, vasculopathy, and progressive fibrosis affecting the skin and internal organs [1]. Vascular injury plays a central role in its clinical expression, manifesting most commonly as Raynaud phenomenon and digital ulceration. Digital gangrene, although recognised, represents the severe end of the vascular spectrum and is reported far less frequently, particularly when it progresses to amputation [2,3].

Although survival of HIV-infected individuals has improved with antiretroviral therapy, the coexistence of HIV and systemic autoimmune conditions remains uncommon [4]. HIV may blunt classical inflammatory expression through profound CD4 lymphopenia, yet paradoxically may also intensify endothelial injury and accelerate tissue damage through chronic immune activation [4]. Published experience describing overlap between SSc and HIV remains limited, and atypical or fulminant manifestations are not well characterised in the literature [2].

This case describes a woman with longstanding untreated SSc who presented with extensive digital gangrene in the context of newly diagnosed advanced HIV infection. The degree of ischaemic destruction was striking and highlights how profound immunosuppression can alter disease trajectory. We present this case to emphasise awareness of this rare overlap, and the diagnostic and clinical implications when severe vascular complications occur in systemic sclerosis.

## Case presentation

**Figure 1:**
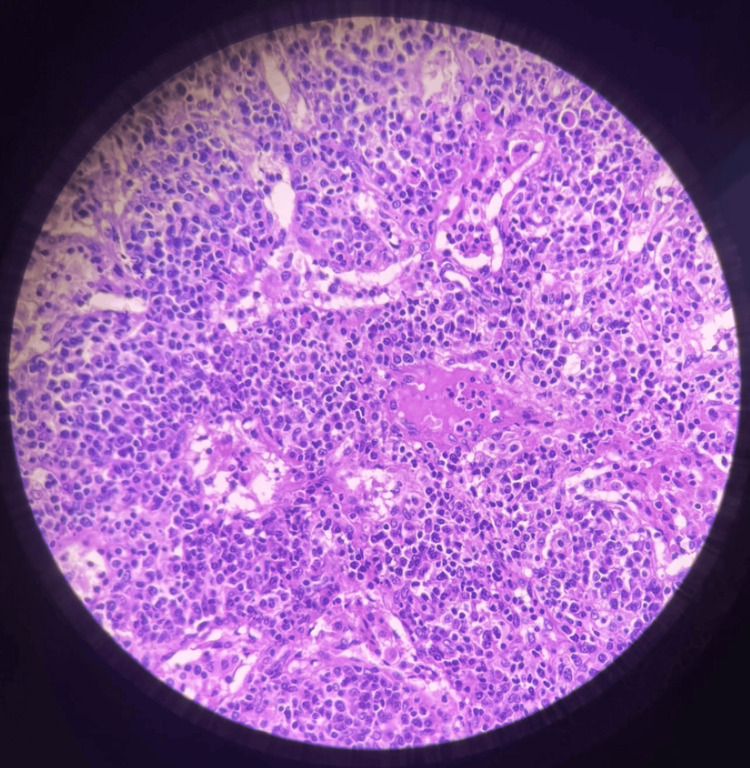
Cervical lymph node biopsy There are numerous well-arranged plasma cells showing nuclear eccentricity, providing an excellent field for assessing the IgG4 count. The fibrosis has a semi-storiform pattern—not fully classic, but strongly suggestive—and there are clear, well-defined fibrous projections and septae. The slide shows minimal fat contamination, which improves readability, and there is also a vessel with a thickened wall that could represent a helpful clue toward phlebitis. Based on histopathology report that the histomorphology could be seen in IgG4-related disease or associated with sclerodermia

A 55-year-old woman with a known five-year history of systemic sclerosis remained untreated (biopsy shown in Figure 1), reportedly due to financial constraints and lack of specialist access. She presented with progressive black discolouration of the fingers and toes, which evolved over several weeks from painful cyanosis to dry gangrene. She also noted increased facial skin tightening, which resulted in microstomia and a characteristic sclerodermoid appearance.

On physical examination, the patient was found to have stable vital signs and was afebrile. The skin appeared tense and shiny over the face and extremities, with evidence of dry, well-demarcated gangrene affecting several digits. Examination of the head, eyes, ears, nose, and throat revealed a sclerodermoid facies characterized by retracted nasal alae and perioral furrowing. Multiple firm, non-tender cervical and axillary lymph nodes were palpable. Examination of the other systems did not reveal any overt signs of acute systemic infection, heart failure, or pulmonary involvement at the time of presentation.

Initial laboratory and imaging investigations were performed to explore the extent of systemic involvement. Table 1 shows the main blood results; Table 2 shows the detailed blood results.

**Table 1:** Main blood investigations Abbreviations: ANA – Antinuclear Antibody; Anti-Scl-70 – Anti–Topoisomerase I Antibody; ESR – Erythrocyte Sedimentation Rate; CRP – C-Reactive Protein; HIV – Human Immunodeficiency Virus; CD4 – Cluster of Differentiation 4.

Category	Test	Result	Reference Range / Interpretation
Autoimmune panel	Anti-Scl-70 ANA	Positive Positive	Negative Negative
Inflammatory markers	ESR	Elevated (115 mm/hr)	< 20 mm/hr (female)
Inflammatory markers	CRP	Elevated (162 mg/L)	< 10 mg/L
HIV serology	HIV antibodies	Positive (confirmed by Western blot)	Negative
HIV serology	CD4 count	72 cells/mm³	500–1,500 cells/mm³
HIV serology	HIV viral load	>100,000 copies/mL	Undetectable in treated individuals

**Table 2:** Detailed blood investigations Abbreviations: WBC – White Blood Cell; Hb – Hemoglobin; Plt – Platelet Count; ESR – Erythrocyte Sedimentation Rate; CRP – C-Reactive Protein; BUN – Blood Urea Nitrogen; Cr – Creatinine; Na – Sodium; K – Potassium; Ca – Calcium; P – Phosphate; Mg – Magnesium; Alb – Albumin; LDH – Lactate Dehydrogenase; LFTs – Liver Function Tests; PT – Prothrombin Time; INR – International Normalized Ratio; PTT – Partial Thromboplastin Time; Fe – Iron; TIBC – Total Iron-Binding Capacity; ANA – Antinuclear Antibody; Anti-Scl-70 – Anti–Topoisomerase I Antibody; HBsAg – Hepatitis B Surface Antigen; HCV Ab – Hepatitis C Virus Antibody; HIV – Human Immunodeficiency Virus; CD4 – Cluster of Differentiation 4.

Category	Test	Result	Reference Range / Notes
Hematology	WBC	5.2 ×10³/µL	4.0–10.0 ×10³/µL
Hematology	Hb	8.5 g/dL	12–15 g/dL (female)
Hematology	Plt	253 ×10³/µL	150–400 ×10³/µL
Hematology	ESR	115 mm/hr	< 20 mm/hr (female)
Hematology	CRP	162 mg/L	< 10 mg/L
Biochemistry	BUN	17 mg/dL	7–20 mg/dL
Biochemistry	Cr	1.2 mg/dL	0.6–1.3 mg/dL
Biochemistry	Na	140 mmol/L	135–145 mmol/L
Biochemistry	K	4.5 mmol/L	3.5–5.0 mmol/L
Biochemistry	Ca	8.8 mg/dL	8.5–10.5 mg/dL
Biochemistry	P	3.8 mg/dL	2.5–4.5 mg/dL
Biochemistry	Mg	2.4 mg/dL	1.7–2.4 mg/dL
Biochemistry	Alb	3.0 g/dL	3.5–5.0 g/dL
Biochemistry	LDH	600 U/L	140–280 U/L
Biochemistry	LFTs	Mildly elevated	AST: < 35 U/L; ALT: < 35 U/L
Coagulation	PT	14–15 sec	11–13.5 sec
Coagulation	INR	1.1	0.8–1.2
Coagulation	PTT	37–39 sec	25–35 sec
Iron Studies	Ferritin	253 ng/mL	13–150 ng/mL (female)
Iron Studies	Serum Fe	33 µg/dL	60–170 µg/dL
Iron Studies	TIBC	184 µg/dL	240–450 µg/dL
Serology/Immunology	ANA	Positive (nucleolar pattern)	Negative
Serology/Immunology	Anti-Scl-70	Positive	Negative
Serology/Immunology	HBsAg, HCV Ab	Negative	Negative
HIV-specific	HIV antibody	Reactive, confirmed	Negative
HIV-specific	CD4 count	72 cells/mm³	500–1,500 cells/mm³
HIV-specific	HIV viral load	>100,000 copies/mL	Undetectable in treated individuals

**Figure 2:**
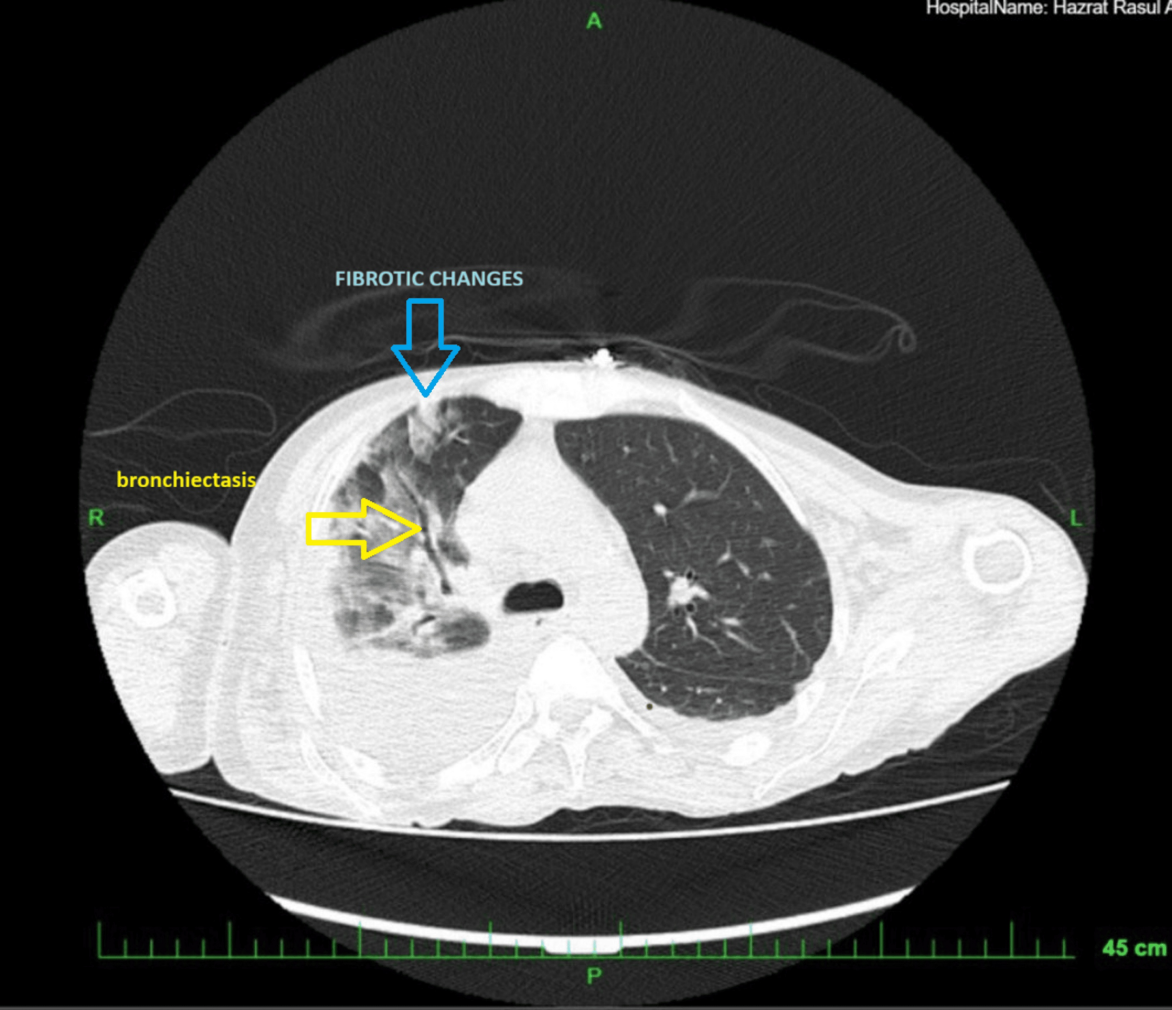
Computed Tomography (CT) scan showing bronchiectasis and fibrotic changes in the right lung.

Doppler ultrasound revealed poor distal perfusion, indicating the presence of microvascular compromise. A CT scan of the neck, chest, abdomen, and pelvis demonstrated generalized lymphadenopathy, while the chest CT specifically showed cylindrical bronchiectasis and subtle fibrotic changes in the right upper lobe (Figure 2). Histopathological examination of the lymph node biopsy revealed chronic inflammatory changes consistent with systemic sclerosis, with no evidence of malignancy.

**Figure 3:**
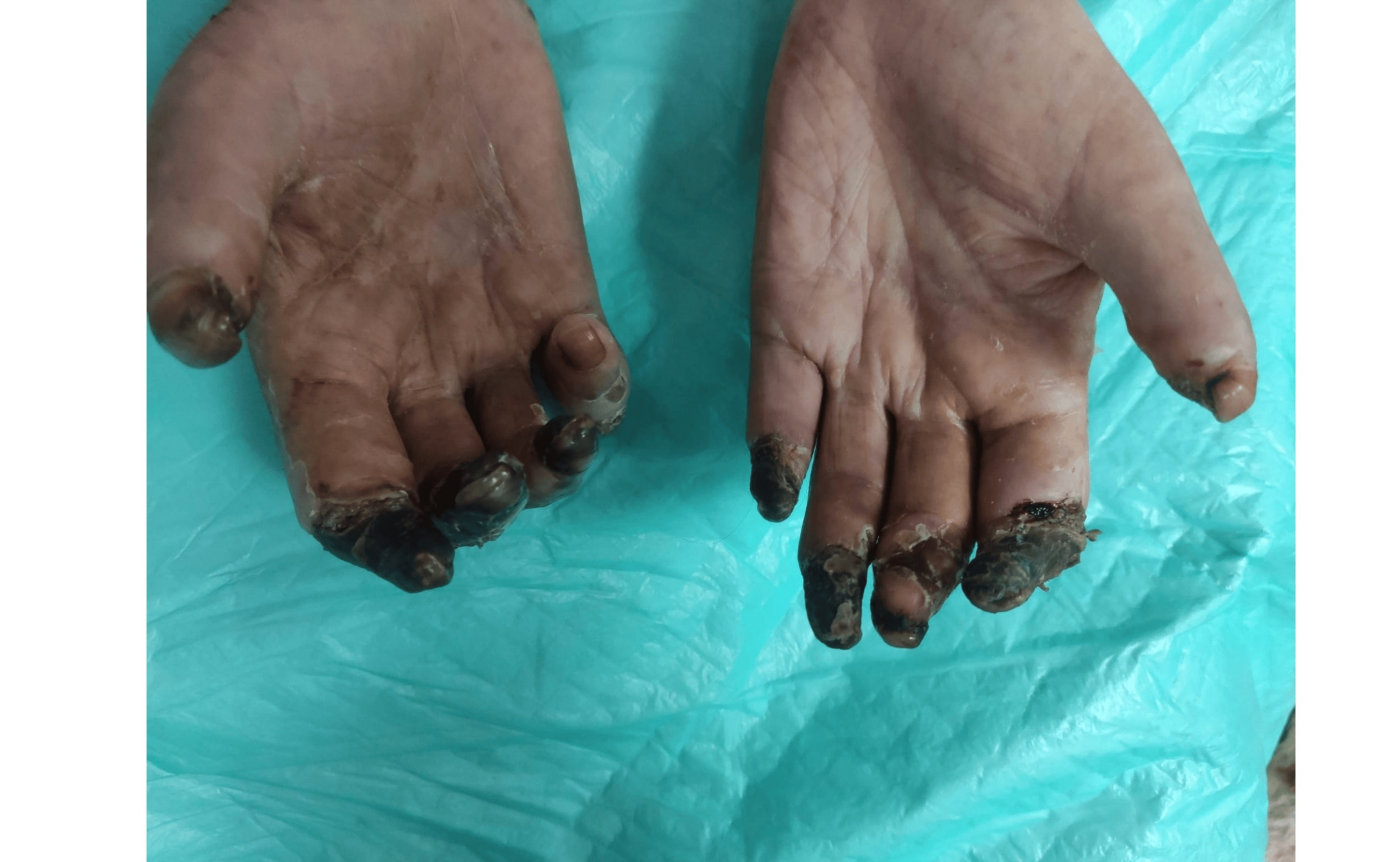
Severity of hand digital gangrene

**Figure 4:**
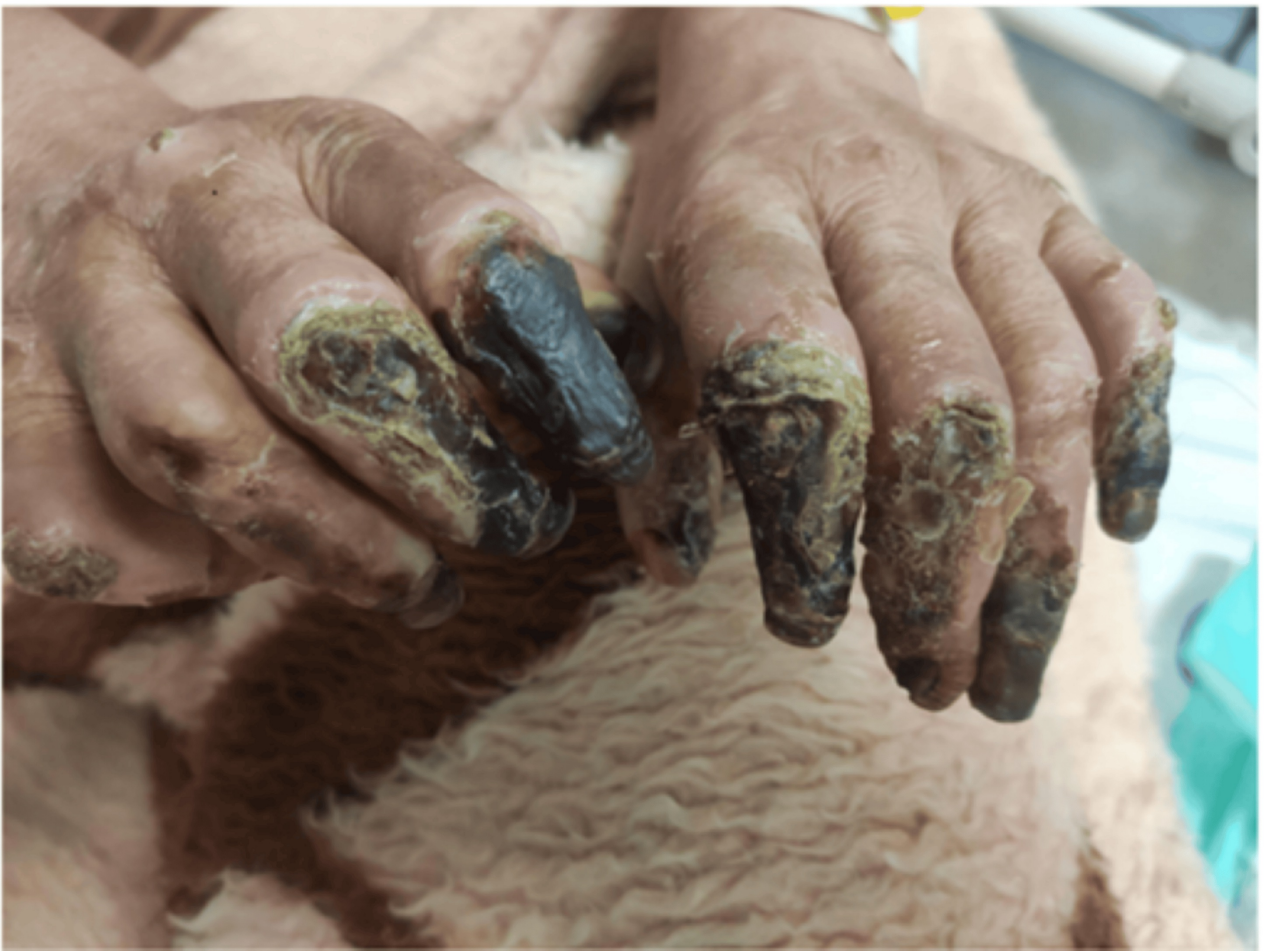
Severity of hand digital gangrene

**Figure 5:**
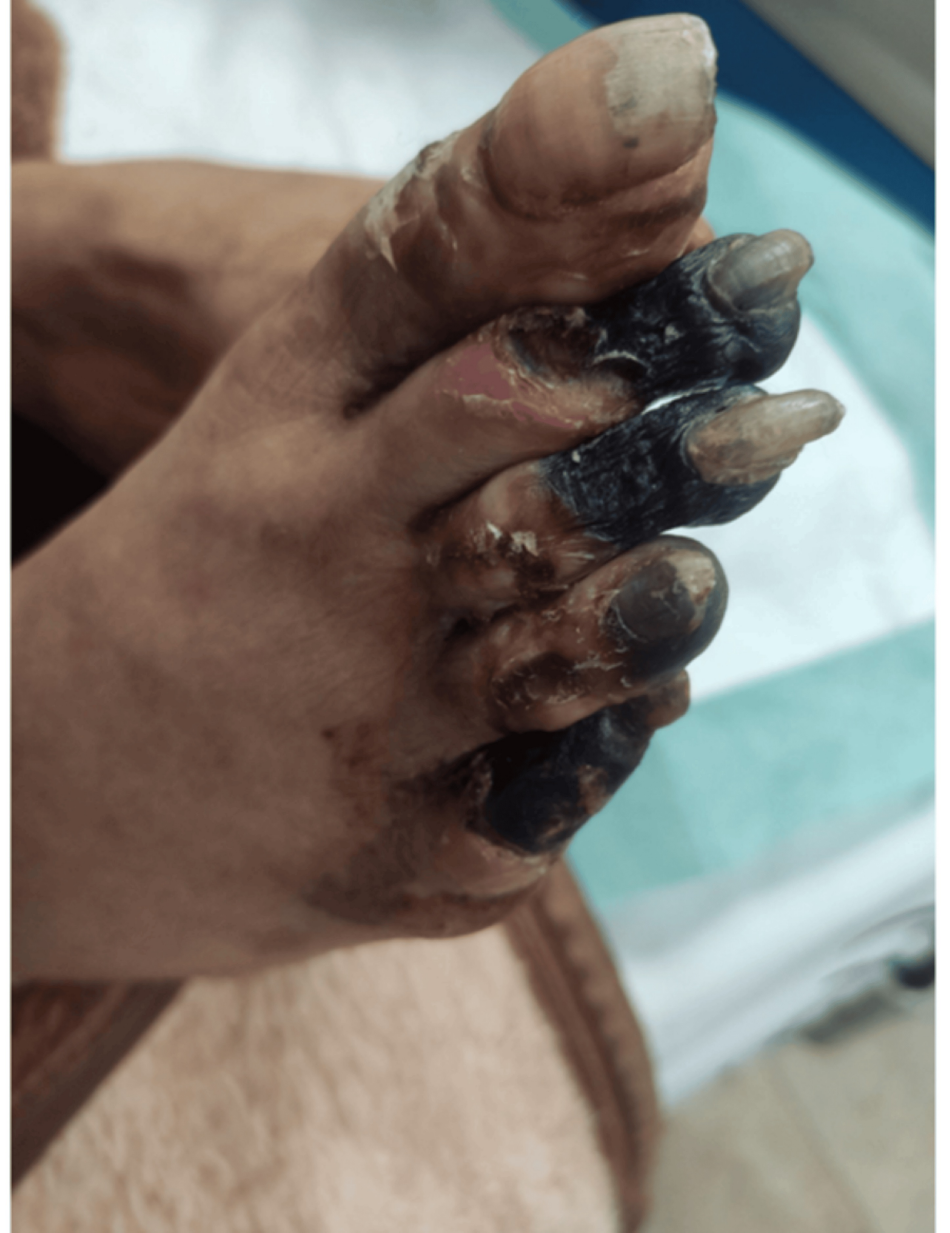
Lower limb toes gangrene

**Figure 6:**
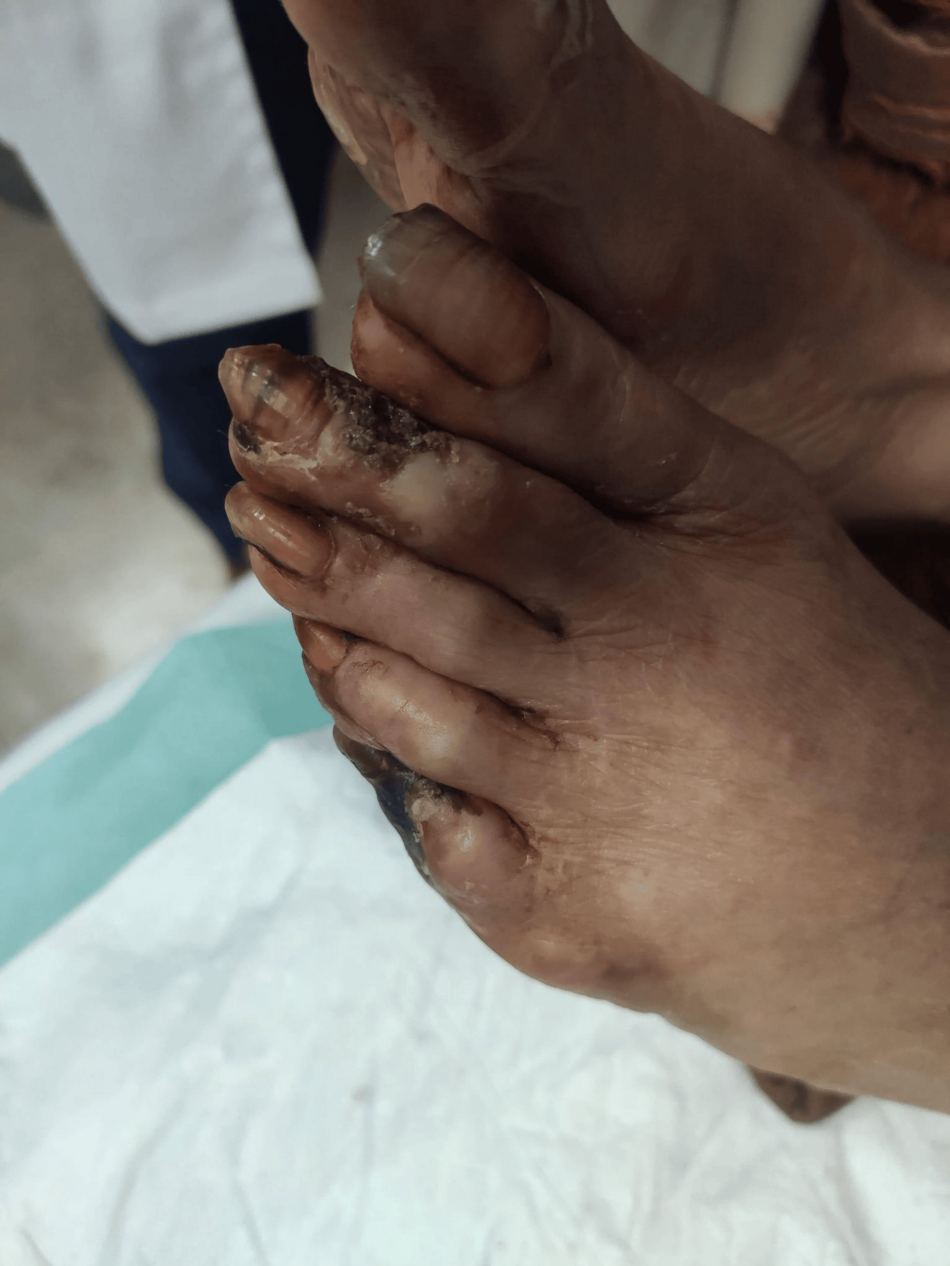
Lower limb toes gangrene

Owing to irreversible ischemic necrosis, the patient underwent amputation of the right toes. She was then transferred to the infectious diseases service, where antiretroviral therapy (bictegravir/emtricitabine/tenofovir) and Pneumocystis jirovecii pneumonia (PCP) prophylaxis were initiated. Vasodilator treatment, including calcium channel blockers and intravenous prostacyclin analogs, was initiated to address the ongoing digital ischemia. Empirical antibiotics were administered perioperatively. Immunosuppressive therapy was deferred until partial immune reconstitution was achieved. She was discharged in stable condition with follow-up arranged in rheumatology and infectious disease clinics. Figures 3-6 show the severity of the gangrene.

## Discussion

SSc is a multisystem autoimmune disorder characterized by vasculopathy, immune dysregulation, and progressive fibrosis of skin and internal organs. Digital gangrene in systemic sclerosis is a rare but severe complication that reflects advanced microvascular damage and chronic endothelial dysfunction [1]. Vascular complications are frequent, with Raynaud’s phenomenon and digital ulceration being common, but frank gangrene is unusual and typically reflects severe vascular compromise [2]. Untreated SSc leads to progressive fibrosis and vascular narrowing, increasing the risk of digital ulcers and critical ischemia. Amputation has been reported in up to 11% of patients with refractory digital ulcers [3]. The present case demonstrates advanced digital ischemia in a patient with untreated SSc, compounded by advanced HIV infection. This unusual association emphasizes the need for vigilance in evaluating aggressive or atypical presentations. The coexistence of HIV and autoimmune disease is uncommon. HIV infection can attenuate some autoimmune manifestations due to profound CD4 lymphopenia, yet paradoxically may amplify vascular injury through chronic immune activation [4]. In this patient, profound immunosuppression likely masked inflammatory features while accelerating tissue loss. Generalized lymphadenopathy raised concerns for malignancy or opportunistic infection, but biopsy revealed chronic autoimmune inflammatory changes. Such overlap poses diagnostic challenges, as features of HIV and SSc may intersect [2]. HIV-associated endothelial dysfunction, along with cytokine dysregulation, may accelerate ischemia in SSc [1]. Furthermore, socioeconomic barriers delaying access to specialist care increase the risk of irreversible complications. Our case illustrates how untreated SSc, combined with HIV-related immune dysfunction, can lead to catastrophic digital necrosis [3]. Priority should be given to antiretroviral therapy with immune reconstitution before considering disease-modifying drugs [5]. In our patient, bictegravir/emtricitabine/tenofovir and prophylaxis for PCP were initiated. Vasodilators, including calcium channel blockers and prostacyclin analogs, are recommended for digital ischemia [6], although evidence in HIV-positive patients is lacking. Treatment requires individualized planning and close collaboration between rheumatology and infectious disease teams [7]. Key clinical lessons emerge. First, severe ischemic complications in SSc should prompt evaluation for secondary contributors, including infection, malignancy, and toxins [8]. Second, atypical or fulminant presentations must trigger suspicion for immunodeficiency. Third, optimal management of overlapping autoimmune and infectious disease requires balancing disease control with infection risk [9-11]. Early diagnosis and multidisciplinary care can reduce morbidity and prevent irreversible outcomes. Clinicians must remain vigilant for atypical disease trajectories in immunocompromised patients, as profound immunodeficiency may both attenuate and amplify autoimmune manifestations in paradoxical ways [12]. Effective management therefore requires careful balance between disease control and infection risk, supported by coordinated multidisciplinary care [13]. In summary, this case highlights the destructive vascular potential of untreated systemic sclerosis in the setting of advanced HIV infection. Awareness of such rare overlap syndromes may facilitate earlier recognition of hidden infections, improve diagnostic accuracy, and guide safe, tailored therapeutic strategies [13,14].

## Conclusions

This case illustrates the importance of evaluating severe ischemic manifestations in systemic sclerosis for underlying or concurrent infectious etiologies, particularly in immunocompromised patients. Early diagnosis and multidisciplinary management are essential for preventing irreversible complications in such complex clinical scenarios.
